# Hints and Improvements in the Practice of Medicine and Surgery

**Published:** 1799-11

**Authors:** 


### HINTS AND IMPROVEMENTS IN THE PRACTICE OF MEDICINE AND SURGERY.

### *To the Editors of the Medical and Phyſical Journal.*

GENTLEMEN,

ONE of your readers has ſolicited communications reſpecting the late introduction of yeaſt into the practice of medicine; ſhould you, therefore, deem that the following remarks may have a tendency to induce others to inveſtigate a practice which appears to be at leaſt harmleſs, if not actively beneficial to thoſe who ſuffer under the languor and wearisomeneſs of fever, you will, by their inſertion, much oblige one who has long admired your valuable labours.

During three months of laſt year, I was called upon in routine, as one of the phyſicians to our Diſpenſary, to attend the Work-houſe of this town, among the children of which a fever had broken out to an alarming degree. Being encouraged by previous ſucceſs, yeaſt was ordered to be adminiſtered in doſes of from two tea ſpoons full to a meat ſpoon full three or four times a day; at the ſame time, the uſual treatment obſerved with reſpect to the other invalids was extended to theſe. In a few days, on the compariſon, I had the pleaſure to obſerve ſome of our little patients much benefited by it; and was particularly ſtruck with the ſpeedy convaleſcence of one, whoſe ſymptoms had been moſt diſtreſſingly ſtaticnary.

The fevers of theſe children had, in ſeveral inſtances, been accompanied with a very remarkable dilatation of the pupils, and an inirritability of the iris to the ſtimulus of light, nearly approaching to that of amauroſis. In other inſtances, the embarraſſing ſymptom of local inflammation proved conſiderably alarming. In both deſcriptions of patients this practice appeared ſerviceable; in the former of which, the return of the iris to its natural ſtate, together with ſomewhat more of the healthy luſtre of the eye in general, were among the firſt ſymptoms of convaleſcence; this even took place before the commencement of appetite, or entire abatement of thirſt. In one patient, the retina became came too senſible to light. Some of theſe young ſufferers, though at firſt averſe to its adminiſtration, after a few doſes reminded the nurſe of it, as though apprehenſive leſt it ſhould be omitted. A conſpicuous advantage gained, was, that anodyne draughts became now ſuperfluous, the irritability of the ſyſtem being diminiſhed. In thoſe fevers accompanied with topical inflammation, on the whole, advantage reſulted from it. Yet, in one caſe, the inflammatory ſymptoms, which were pulmonary, advanced ſo rapidly as to cauſe me to decline its continuance, till theſe ſymptoms were ſubdued ; afterwards it was given without inducing a return of inflammation. In this caſe, the digitalis in tincture, combined with a few drops of T. opii, was of deciſive benefit. That the increaſe of theſe ſymptoms had any connexion with the uſe of the yeaſt, a ſolitary fact will not warrant the concluſion.

The bowels were not affected by it, nor did the pulſe alter for ſome days ; yet I thought the cutaneous heat was relieved, though of this I ſpeak with diffidence, as cold ablutions were ſtrictly attended to, where requiſite ; a practice which cannot be too highly extolled, and which has been ſo ably treated of in a late very elegant and truly valuable publication.

In the firſt fever wherein I adopted this practice, the patient, a young woman of 19, of ruddy complexion, naturally plethoric, and liable to inflammatory ailments, was informed of its origin, and that it was far from being commonly in uſe, but that it would at leaſt be harmleſs. The family poſſeſſing more good ſenſe and energy of mind than generally happens to the uneducated, made none of thoſe frivolous objections ſo often met with, where a patient is truſted with the knowledge of the medicine preſcribed. She had taken the infection from her ſiſter, whoſe caſe had been truly deplorable, and nearly deſperate. The ſiſters were obliged to lie in one bed, and their diet had been latterly poor, as the mother was afflicted with dyſentery during and prior to the ſickneſs of her two fatherleſs daughters. Being patients belonging to the Diſpenſary, and the emetic which was ordered previouſly to any other medicine being loſt, the circumſtance was concealed till after recovery. She dreading a ſituation ſimilar to that of her ſiſter, whom ſhe had watched night and day with all the aſſiduity of affection, took yeaſt to the amount of a meat ſpoon full four, five, and ſometimes ſix times a day : Her natural colour returned in about five days ; and, to my ſurprize, I found her affected with a ſlight, though complete cynanche tonſillaris ; on which I omitted the medicines, and ordered merely gargles with an external ternal liniment; and in a few days ſhe was again able to return to the duties of a nurſe to her ſiſter, which the mother, though now recovered, was ſcarcely equal to perform. Some have complained much of an uneaſy ſenſe of diſtention ariſing from the medicine, a circumſtance which has cauſed more than one patient to relinquiſh its uſe, as did this perſon on the laſt day of her taking it. I ought to add, that in ſome inſtances diſappointment has attended its exhibition, though, ſave in hoſpitals, we cannot rely on the adoption of a practice which popular prejudice ridicules, or ſtarts at. In ſome inſtances I have no doubt but deception has been practiſed. In a caſe of cynanche maligna, as a gargle, and internally adminiſtered, I thought it aided other remedies.

The caſe of the young woman recorded, if confirmed by future obſervations, will be replete with information.

ROBERT LEWIN.

